# Sodium pyruvate attenuates oxidative stress, lowers ox-LDL and ox-LDL/LDL-P, and mitigates inflammation in diabetic atherosclerotic rats

**DOI:** 10.1515/biol-2025-1318

**Published:** 2026-05-29

**Authors:** Ahlam Mohamed Yusuf, Xiehui Chen, Minmin Weng, Zhenzhen Su, Wei Hu, Nan Wu, Jing Xu, Binsheng Chen, Lishan Wu, Wei Sun, Xiaomin Chen, Zhanke Wang

**Affiliations:** Department of Cardiology, First Affiliated Hospital of Ningbo University, Ningbo 315010, China; Ningbo Key Laboratory for Precision Detection of Lipoprotein Subfractions by VAP, Ningbo Meikang Shengde Medical Laboratory, Ningbo 315105, China; Department of Geriatrics, Shenzhen Longhua District Central Hospital, Shenzhen 518110, China; Department of Clinical Laboratory, The Fourth Affiliated Hospital of Nanchang University, Nanchang 330002, China; College of Biological & Environmental Sciences, Zhejiang Wanli University, Ningbo 315105, China; Department of Ultrasound Medicine, Ningbo Yinzhou Hospital of Traditional Chinese Medicine, Ningbo 315105, China

**Keywords:** diabetic atherosclerosis, ox-LDL, ox-LDL/LDL-P, inflammation, sodium pyruvate, oxidative stress

## Abstract

This study aimed to investigate the protective effects of sodium pyruvate against diabetic atherosclerosis in rats by reducing oxidative stress, ox-LDL levels, and inflammatory responses. Wistar rats were randomly divided into four groups: normal control (NC), diabetes mellitus (DM), diabetic atherosclerosis (DA), and diabetic atherosclerosis treated with sodium pyruvate (DA + SP). Serum levels of malondialdehyde (MDA), superoxide dismutase (SOD), remnant-like particle cholesterol (RLP-C), ox-LDL, low-density lipoprotein particle (LDL-P), the ox-LDL/LDL-P ratio, high-sensitivity C-reactive protein (hs-CRP), and interleukin-6 (IL-6) were measured. Compared with NC and DM groups, DA rats exhibited increased oxidative stress, lipoprotein abnormalities, and inflammation, evidenced by elevated MDA, RLP-C, ox-LDL, LDL-P, ox-LDL/LDL-P ratio, hs-CRP, and IL-6, along with reduced SOD levels. Sodium pyruvate treatment significantly decreased MDA, ox-LDL, ox-LDL/LDL-P ratio, hs-CRP, and IL-6, while increasing SOD activity compared with the DA group. No significant differences in RLP-C and LDL-P were observed between DA and DA + SP groups. Sodium pyruvate alleviates oxidative stress and inflammation and reduces ox-LDL-related indices, suggesting a protective role in diabetic atherosclerosis.

## 1 Introduction

Nutritional excess and adverse lifestyle habits readily induce dyslipidemia, exemplified by heightened oxidized low-density lipoprotein (ox-LDL) concentrations. Dyslipidemia subsequently promotes atherosclerosis formation, a persistent inflammatory condition characterized by endothelial dysfunction, lipid deposition in the vascular intima, recruitment of inflammatory cells, and localized production of cytokines [1]. Atherosclerosis is associated with a high risk and mortality rate for arteriosclerotic cardiovascular disease (ASCVD) [2], 3]. Among the numerous risk factors for ASCVD, diabetes is a critical contributor, as it is accompanied by oxidative stress, dyslipidemia and inflammation [4], [5], [6], [7], [8], [9]. Compared to healthy individuals, diabetic patients face an approximately 2–4 times higher risk of developing ASCVD [10].

Diabetes is often accompanied by dyslipidemia, characterized by elevated levels of lipoprotein subfractions [4], 5]. Patients with diabetes also exhibit heightened oxidative stress and inflammation [6], [7], [8], [9]. Oxidative stress in diabetes increases LDL oxidation into ox-LDL. Ox-LDL accelerates atherosclerotic development by inducing inflammatory responses [11], 12]. Despite current approaches to diabetes management prioritizing meticulous control of systemic risk factors like elevated blood sugar and abnormal lipid levels, they fall short when it comes to addressing the oxidative stress and inflammation that diabetes worsens. This shortfall underscores the urgent requirement for treatments that zero in on specific pathological mechanisms rather than just treating symptoms. Sodium pyruvate is known for its significant antioxidant and anti-inflammatory capabilities [13], 14] and enhances protection for organs such as the heart and brain by improving hemodynamics and vascular permeability [15], 16].

Diabetic patients with atherosclerosis exhibit oxidative stress and inflammation [17]. However, whether sodium pyruvate exerts anti-inflammatory effects in diabetic atherosclerosis by alleviating oxidative stress and reducing ox-LDL has not been reported domestically and internationally. Although *in vitro* experiments have demonstrated that ox-LDL can induce atherosclerotic inflammatory responses [11], there is a lack of *in vivo* experimental evidence for ox-LDL-induced atherosclerosis, and no *in vivo* studies have reported the induction of atherosclerotic inflammatory responses by intravenous injection of ox-LDL in healthy organisms.

Therefore, we employed diabetic atherosclerotic rats as the study subjects and utilized sodium pyruvate to specifically reduce ox-LDL without decreasing other lipoprotein subfractions such as LDL-P and RLP-C, in order to observe the effects of solely lowering ox-LDL on inflammatory factors and the amelioration of atherosclerosis in diabetic atherosclerotic rats. On one hand, this provides *in vivo* experimental evidence that ox-LDL induces inflammatory responses in diabetic atherosclerotic rats, validating the *in vitro* findings that ox-LDL can promote atherosclerotic inflammation; on the other hand, it offers novel insights into sodium pyruvate as an antioxidant, by reducing ox-LDL, as a potential agent for the prevention and treatment of diabetic atherosclerosis.

## 2 Materials and methods

### 2.1 Animal subjects and experimental procedures

Eighty male Wistar rats, weighing between 240 and 290 g, were housed under controlled environmental conditions with a 12-h light/dark cycle, temperature of 22 ± 2 °C, and humidity of 55 ± 5 % after procurement from Zhejiang Weitonglihua Experimental Animal Technology. The rats were randomly assigned to two groups: a normal control group (NC, *n* = 20) and a diabetes model group (*n* = 60). The 60 rats were intraperitoneally injected with streptozotocin (35 mg/kg, Coibo Biotechnology Co., Ltd, Shanghai, China) to destroy pancreatic β cells. After 72 h, blood samples were drawn from the rats for assessment of plasma glucose levels. According to the literature [18], successful induction of diabetes in the rats was confirmed by a fasting plasma glucose level exceeding 7.8 mmol/L. Next, 20 rats were randomly selected from the diabetes model rats as the diabetes mellitus group (DM group, *n* = 20) with normal feeding. And in order to establish the diabetic atherosclerosis model, 40 rats with established diabetes were randomized to undergo a 12-week induction regimen consisting of a high-fat diet combined with intraperitoneal injections of vitamin D_3_ (6,000,00 IU/kg) [19]. The obvious pathological changes of atherosclerosis in the rat abdominal aorta indicated that the diabetic atherosclerosis model was successfully created. Then these 40 diabetic atherosclerosis model rats were randomly divided into a diabetic atherosclerosis with sodium pyruvate treatment group (DA + SP group, *n* = 20) and a diabetic atherosclerosis group (DA group, *n* = 20). Sodium pyruvate administration (i.p., 30.0 mg/kg/d, 4 weeks) was assigned to the DA + SP group, while the rats in the DA group received normal saline injections instead.

**Ethical approval:** The research related to animal use has been complied with all the relevant national regulations and institutional policies for the care and use of animals, and has been approved by the Ethics Committee of Ningbo Meikang Shengde Medical Laboratory.

### 2.2 Detection of plasma RLP-C, ox-LDL, LDL-P, hs-CRP, IL-6, MDA and SOD levels

Rats were fasted for 12 h prior to tail vein blood collection. Plasma ox-LDL, IL-6 and MDA levels were determined using the ELISA method (Coibo Biotechnology Co., Ltd., Shanghai, China). Plasma hs-CRP and SOD levels were measured using the enzyme method on the MS2080 fully automated biochemical analyzer (Medicalsystem Biotechnology Co., Ltd., Ningbo, China). Plasma RLP-C and LDL-P levels were quantified using the vertical auto profile (VAP) method on the MS-V600 lipoprotein subfraction detection system and MS-V800 lipoprotein particle detection system (Medicalsystem Biotechnology Co., Ltd., Ningbo, China).

### 2.3 Pathological change of atherosclerosis in rat abdominal aortas

After euthanasia by CO_2_ inhalation, rat abdominal aortas were made into paraffin-embedded sections and stained with hematoxylin-eosin. Firstly, the rat abdominal aortas were fixed in 10 % neutral formaldehyde. After fixation, the tissues underwent standard histological processing, encompassing dehydration through a graded ethanol series, xylene clearing, and final embedding in molten paraffin for block sectioning. To begin the staining process, sectioned tissues underwent deparaffinization in xylene, followed by rehydration in a series of ethanol solutions with progressively decreasing concentrations. For histological staining, sections were first stained with hematoxylin, differentiated, and then counterstained with eosin. To conclude the staining process and prepare the sections for archival preservation, they were cleared in xylene and subsequently mounted under a permanent neutral balsam medium. Finally, pathological changes of atherosclerosis in rat abdominal aortas were observed under a microscope.

### 2.4 Data analysis methods

The study data were statistically analyzed utilizing SPSS version 21.0 from IBM Corp. (Armonk, NY, USA). Intergroup comparisons of these parameters (ox-LDL, RLP-C, LDL-P, the ox-LDL/LDL-P ratio, hs-CRP, IL-6, MDA and SOD) were conducted using one-way ANOVA. Tukey’s HSD test to compare the means between two groups. *P* < 0.05 was considered statistically significant.

## 3 Results

### 3.1 Changes in rat plasma MDA and SOD levels

**Figure 1:**
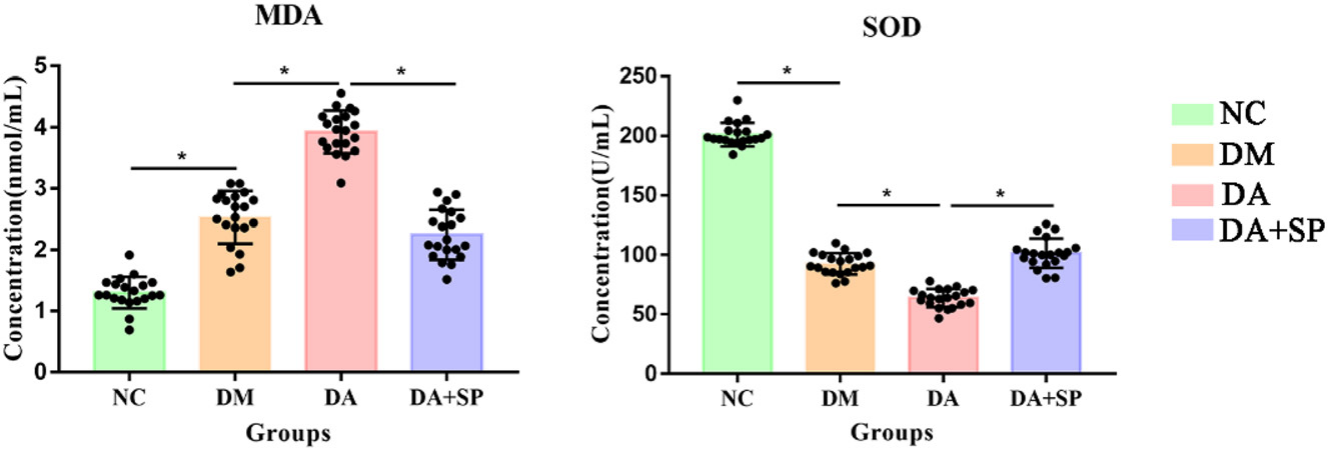
The differences in MDA and SOD levels among the groups. NC: normal control, DM: diabetes mellitus, DA: diabetic atherosclerosis, DA + SP: diabetic atherosclerosis with sodium pyruvate treatment, *n* = 20 per group, *P < 0.05.

Plasma MDA levels showed a step-wise elevation among the experimental groups, with the lowest concentrations in the NC group, intermediate in the DM group, and the highest in the DA group (*P* < 0.05). Conversely, plasma SOD levels showed a step-wise decline among the experimental groups, with the highest concentrations in the NC group, intermediate in the DM group, and the lowest in the DA group (*P* < 0.05). In contrast, the DA + SP group demonstrated significantly lower MDA levels and higher SOD levels compared to the DA group (*P* < 0.05) (Figure 1).

### 3.2 Changes in rat plasma RLP-C, ox-LDL, LDL-P and ox-LDL/LDL-P

**Figure 2:**
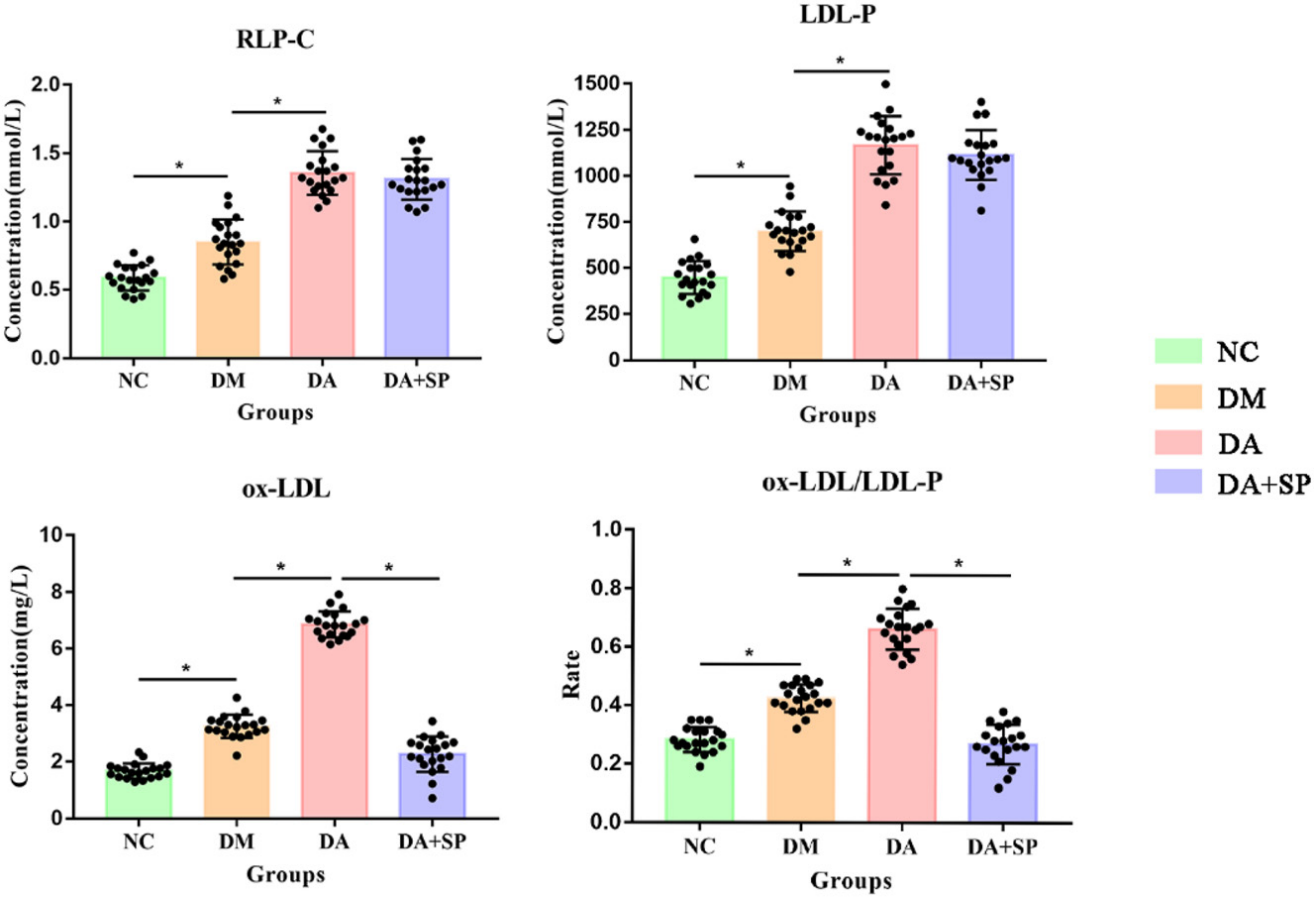
The differences in RLP-C, LDL-P, ox-LDL, and ox-LDL/LDL-P among the groups. NC: normal control, DM: diabetes mellitus, DA: diabetic atherosclerosis, DA + SP: diabetic atherosclerosis with sodium pyruvate treatment, *n* = 20 per group, *P < 0.05.

Plasma RLP-C, LDL-P, ox-LDL, and the ox-LDL/LDL-P ratio showed a step-wise elevation among the experimental groups, with the lowest concentrations in the NC group, intermediate in the DM group, and the highest in the DA group (*P* < 0.05). Additionally, the DA + SP group exhibited a marked reduction in plasma ox-LDL and ox-LDL/LDL-P compared to the DA group (*P* < 0.05). Sodium pyruvate supplementation did not significantly alter plasma RLP-C or LDL-P levels compared to the DA group (Figure 2).

### 3.3 Changes in rat plasma hs-CRP and IL-6 levels

**Figure 3:**
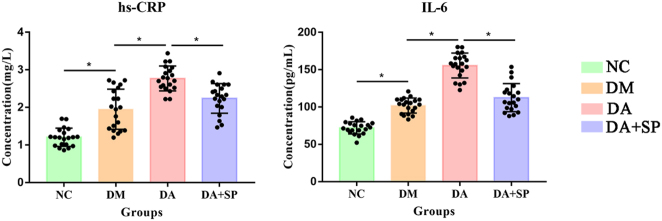
The differences in hs-CRP and IL-6 levels among the groups. NC: normal control, DM: diabetes mellitus, DA: diabetic atherosclerosis, DA + SP: diabetic atherosclerosis with sodium pyruvate treatment, *n* = 20 per group, *P < 0.05.

The plasma hs-CRP and IL-6 levels were significantly higher in the DA group than in the DM group, and significantly higher in the DM group than in the NC group (*P* < 0.05). Additionally, the DA + SP group exhibited a marked reduction in plasma hs-CRP and IL-6 levels compared to the DA group (*P* < 0.05) (Figure 3).

### 3.4 Pathological changes in rat abdominal aortas

**Figure 4:**
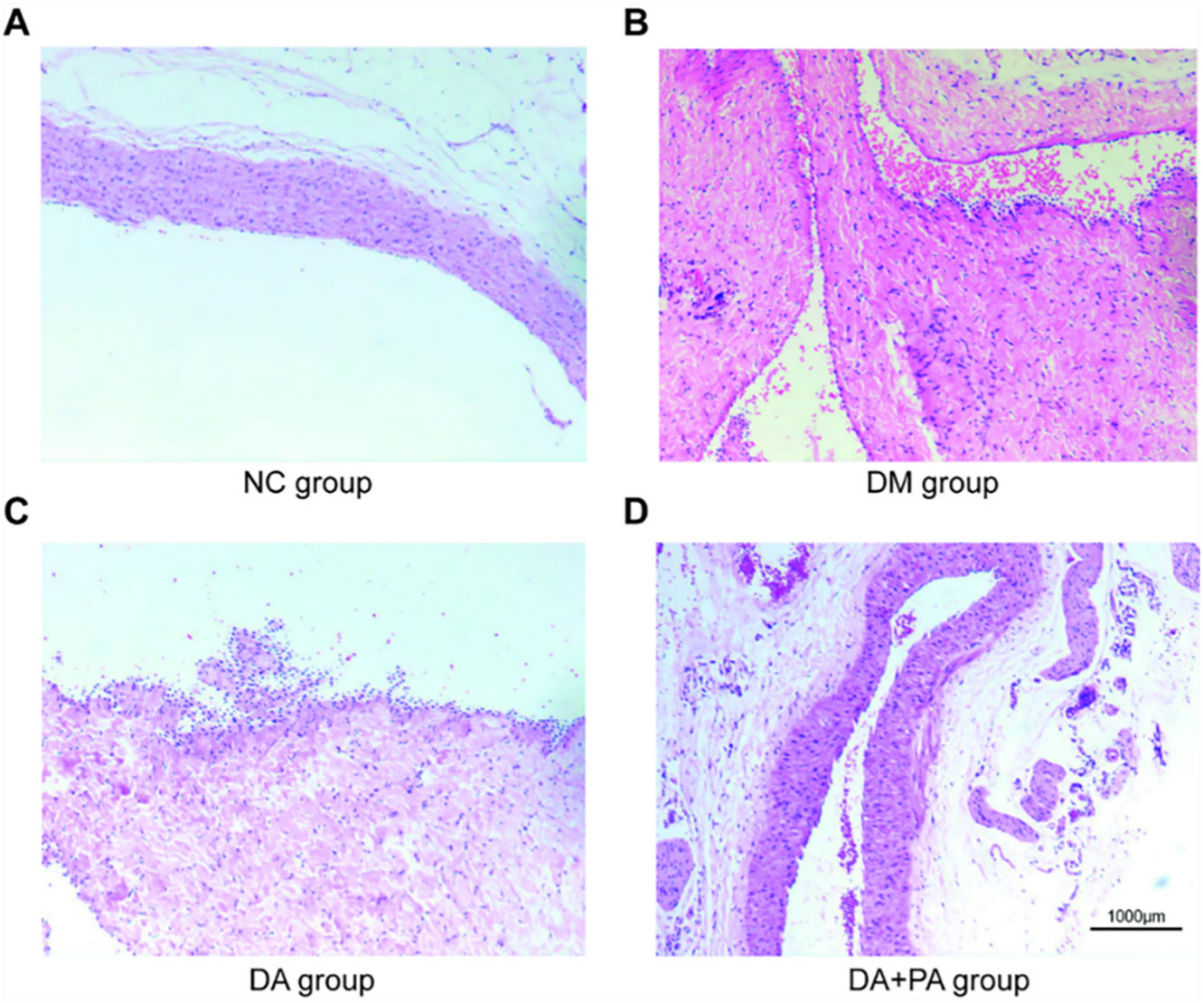
HE staining in each group. (A) NC group. (B) DM group. (C) DA group. (D) DA + SP group. Scale bar: 1,000 μm.

The abdominal aortic vessel wall of the DM group showed mild thickening, with visible folds and protrusions, disorganized arrangement of vascular endothelial cells, and a small number of inflammatory cells, which were absent in the NC group. The DA group exhibited significant thickening of the abdominal aortic wall, structural abnormalities, noticeable folds and prominent protrusions, disordered arrangement of vascular endothelial cells, and abundant adipocytes, foam cells, and inflammatory cells. None of these pathologies was observed in the NC controls. The pathological features in the DA + SP group were significantly mitigated relative to the DA group, showing a largely normal abdominal aortic wall structure with smooth margins, clear architecture, nearly normal arrangement of vascular endothelial cells, and only a small number of adipocytes, foam cells, and inflammatory cells. These morphological improvements collectively indicate an amelioration of atherosclerotic inflammatory response in the abdominal aorta (Figure 4A–D).

## 4 Discussion

Diabetes mellitus is an independent risk factor for atherosclerosis, and vitamin D3 has been shown to accelerate atherosclerosis [20]. This rationale underpins our experimental design: first establishing a diabetic model, then inducing atherosclerosis via a high-fat diet and intraperitoneal injection of vitamin D3. Due to oxidative stress and inflammatory responses, diabetes is prone to developing atherosclerosis [21], [22], [23], [24], [25], [26], [27], [28]. Our findings demonstrated that the plasma ox-LDL and MDA levels were significantly elevated, while the SOD levels were significantly reduced in diabetic rats. This likely reflects the presence of oxidative stress in diabetes. Diabetic rats with atherosclerosis exhibited even higher plasma ox-LDL and MDA levels, and lower SOD levels. This suggests the high-fat diet exacerbates disorders of lipid metabolism and oxidative stress in diabetic rats. It further indicates that under conditions of impaired glucose metabolism, a high-fat diet poses a greater hazard to vascular health. The combination of diabetes and poor dietary habits significantly increases susceptibility to ASCVD.

Atherosclerosis is a common complication of diabetes. The transformation of macrophages into foam cells, triggered by the engulfment of ox-LDL, is a pivotal step in atherosclerotic lesion development [29]. Ox-LDL activates receptors on the macrophage surface, elevating levels of cytokines and chemokines, thereby recruiting more circulating immune components and amplifying the local inflammatory response [11], 12]. Our study found that plasma levels of ox-LDL, hs-CRP and IL-6 were elevated in diabetic rats. Notably, diabetic rats with atherosclerosis exhibited even higher plasma levels of ox-LDL, hs-CRP and IL-6.

Low-density lipoprotein cholesterol (LDL-C) refers to the cholesterol concentration within LDL particles, while LDL-P encompasses the LDL particle concentration, which is composed of lipids and apolipoproteins. Ox-LDL represents the concentration of oxidatively modified LDL particles. LDL-C has limitations in reflecting ASCVD risk and cannot fully capture the residual risk of ASCVD [30]. Consequently, our research has shifted focus to LDL-P, ox-LDL, and the ox-LDL/LDL-P ratio. Early studies indicated that elevated LDL-P is associated with increased coronary artery calcification and coronary heart disease (CHD) in metabolic syndrome patients [31], 32]. However, recent investigations have presented conflicting perspectives regarding the direct correlation between LDL-P and CHD [33]. Our findings in diabetic rats demonstrate significantly elevated plasma levels of LDL-P, ox-LDL, and ox-LDL/LDL-P, with these parameters being further elevated in diabetic rats with atherosclerosis. These results suggest that oxidative modification of LDL (forming ox-LDL) and an elevated ox-LDL/LDL-P ratio are critical in inducing vascular atherosclerosis and subsequent ASCVD. Non-oxidized LDL, however, is internalized by the LDL receptor, mediates negative feedback regulation, cannot activate scavenger receptors and downstream inflammatory pathways such as TLR4/NF-κB, and is essentially incapable of causing atherosclerosis. Under the condition that the LDL-P level remains unchanged, specifically reducing ox-LDL and the ox-LDL/LDL-P ratio can significantly relieve inflammation and improve atherosclerotic lesions. These findings support, in this rat model, that oxidative modification (ox-LDL) rather than particle number per se may be more closely related to inflammation and lesion burden. The ox-LDL/LDL-P ratio may serve as a novel biomarker for diabetic patients with atherosclerosis, offering a more refined assessment of atherogenic risk than LDL-P alone**.** However, the underlying mechanisms and clinical significance of peripheral blood ox-LDL/LDL-P changes in diabetic atherosclerosis need further investigation.

Pyruvate, as an endogenous substance in the human body, is primarily synthesized from glucose via glycolysis in the cytoplasm and is integral to the metabolism of the three major nutrients. Sodium pyruvate, the sodium salt of pyruvate, exhibits potent antioxidant and anti-inflammatory effects [13], 14], 34]. Our previous study reported that pyruvate exerts antioxidant and anti-inflammatory effects in rats with severe burn-induced multiple organ dysfunction [35]. This study found that in diabetic rats with atherosclerosis treated with sodium pyruvate, plasma SOD levels were elevated, while MDA levels were reduced. Similarly, hs-CRP and IL-6 levels also decreased, consistent with previous findings. Notably, sodium pyruvate treatment led to a reduction in plasma ox-LDL and ox-LDL/LDL-P ratio, though no significant difference was observed in plasma LDL-P levels before and after treatment. Despite these changes, atherosclerosis in the rat abdominal aorta was still ameliorated. This suggests that sodium pyruvate exerts its protective effects through antioxidant mechanisms, by reducing ox-LDL rather than native LDL-P.

New research illustrates and enhances earlier work demonstrating that lipid changes caused by oxidative stress have an important role to play in atherosclerosis (the build-up of plaques in the arteries). Oxygen-containing molecules have also been shown to not only contribute to oxidative stress but also to help oxidise low-density lipoproteins (LDL) and increase the number of foam cells present, which all lead to the formation of more plaques [3]. Oxidised-LDL (or ox-LDL) is now being recognised as an important immunometabolic mediator connecting lipid metabolism with vascular inflammation via activation of endothelial cells and macrophages [12]. Clinical and transitional studies have also shown that elevated levels of ox-LDL are associated with vascular structural damage and increased overall atherosclerotic burden in diabetic patients. In recent years, new therapeutic approaches have been developed to inhibit oxidative stress pathways, instead of the older method of simply trying to reduce total LDL levels; selective reduction in the amount of ox-LDL present and correcting the overall balance between oxidants and antioxidants appears to effectively reduce inflammation and slow disease progression (consistent with the current study). These findings together with those from the present investigation provide additional support for the concept that sodium pyruvate protects against oxidative stress and inflammatory pathways driven by ox-LDL.

These results further emphasize the critical role of ox-LDL and ox-LDL/LDL-P elevation in the development and progression of atherosclerosis *in vivo*, validating the *in vitro* experimental results that ox-LDL induces atherosclerotic inflammation. Our findings support the hypothesis that native LDL-P elevation may not contribute to atherosclerosis, whereas only ox-LDL exhibits pro-atherogenic properties. Our results also suggest that without reducing ox-LDL, merely lowering LDL-P, even when LDL-P is already at low levels, may still lead to elevated ox-LDL due to oxidative stress, potentially resulting in residual ASCVD risk. Our findings indicate that, from a new perspective of reducing oxidative stress and lowering ox-LDL, it provides novel insights for addressing residual ASCVD risk in individuals with normal LDL-P or LDL-C. Future studies should explore optimal dosing strategies and longer treatment durations to evaluate the full therapeutic potential of sodium pyruvate in this complex disease model. Sodium pyruvate is only one of many antioxidants; more antioxidants for reducing ox-LDL in ASCVD patients are also worthy of investigation.

## 5 Conclusions

Diabetic atherosclerotic rats exhibit elevated oxidative stress, increased ox-LDL and ox-LDL/LDL-P, as well as elevated inflammatory response. Sodium pyruvate as an antioxidant, may effectively reduce ox-LDL and ox-LDL/LDL-P levels by reducing oxidative stress, potentially playing a role in anti-inflammatory and improving atherosclerosis in diabetic atherosclerotic rats. However, further research and clinical trials are necessary to explore the potential of sodium pyruvate as a therapeutic agent for atherosclerosis.
